# Case report of an unusual combination of purulent pericarditis and false aneurysm of the ascending aorta

**DOI:** 10.1186/s13019-018-0699-4

**Published:** 2018-01-29

**Authors:** David Meier, Matthias Kirsch, Salah Dine Qanadli, Olivier Muller, Daniel Fishman, Catalina Trana

**Affiliations:** 1 Department of Internal Medicine, University Hospital of Lausanne, Lausanne, Switzerland; 2 Service of Cardiovascular Surgery, University Hospital of Lausanne, Lausanne, Switzerland; 3 Department of Radiology, University Hospital of Lausanne, Lausanne, Switzerland; 4 Service of Cardiology, University Hospital of Lausanne, Lausanne, Switzerland; 5 Service of Emergency Medicine, Riviera-Chablais Hospital, Monthey, Switzerland; 6 Service of Cardiology, Riviera-Chablais Hospital, 1870 Monthey, Switzerland

**Keywords:** Purulent pericarditis, Infected aneurysm, Ascending aorta

## Abstract

**Background:**

Purulent pericarditis is an uncommon entity, which is, in very rare cases, associated to infection of the aorta.

**Case presentation:**

We present the case of a 42-year-old male patient, who was admitted to hospital complaining of tiredness, diarrhea and leg edema. Clinical examination revealed a hypotensive and obviously shocked patient. He was ultimately diagnosed with a rare combination of purulent pericarditis followed by false aneurysm of the ascending aorta. He was successfully treated by surgical pericardial drainage, replacement of the ascending aorta and antibiotics.

**Conclusion:**

Mycotic aneurysms can rarely be associated with purulent pericarditis. Our literature review shows that there are two mechanisms explaining this association and that in most of the published cases infective endocarditis could not be demonstrated.

## Background

Purulent pericarditis has become an uncommon condition since the development of antibiotics. While it was a well-known complication of bacterial pneumonia, it is now much rarer and occurs mostly after thoracic trauma or surgery and in immunocompromised patients. *S. aureus* and *Streptococci* are the commonest identified pathogens.

Infected aneurysm can be caused by direct inoculation during trauma, hematogenous spread, contiguous infection and as vascular phenomenon during bacterial endocarditis. The later forms a subgroup called mycotic aneurysm. Risk factors for mycotic aneurysm are the same as for bacterial endocarditis, with the addition of preexisting aneurysm and atherosclerosis as factors promoting bacterial colonization of the arterial wall. Intracranial arteries are frequently involved, as are leg arteries, while infection of the aorta is rarer but often accompanied by a morphology of pseudo-aneurysm. *Salmonella* species and *Staphylococcus* (especially *S. aureus*) are the most frequently isolated pathogens.

To our knowledge, there are very few cases reporting the combination of these two conditions.

## Case presentation

A 42-year-old man was admitted to a Swiss regional hospital with a complaint of tiredness associated with a 2 weeks’ history of diarrhea, jaundice and 3 days of legs edema. His medical history has shown intravenous drug use, untreated HIV, and advanced liver cirrhosis due to chronic hepatitis C infection. He had been treated the past month for a chronic ulcer of the right ankle.

On initial physical examination, the patient was obviously shocked with somnolence, dehydration, and marbling of the extremities. Cutaneous status was remarkable for presence of sequels of multiples intravenous injections, necrosis of 3 toes and persistence of the right ankle’s ulcer.

**Fig. 1:**
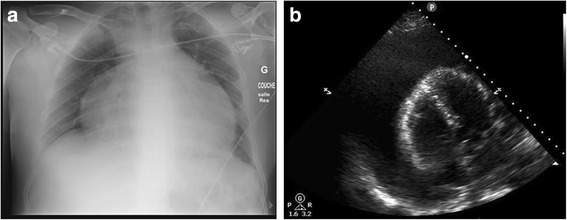
**a** Chest X-ray showing enlarged cardiac silhouette; **b** POCUS picture revealing a very large pericardial effusion

Vitals signs were the following: BP 116/79 mmHg, HR 152 bpm, temp 36.8 °C and oxygen saturation 93%, while breathing ambient air. Arterial blood gases were rapidly obtained and showed lactic acidosis with a pH of 7.28 and a concentration of lactates of 8 mmol/l. ECG showed sinus tachycardia with electrical alternans and the chest X- Ray revealed a massively enlarged cardiac silhouette (Fig. 1a). A point of care ultrasound (POCUS) was then performed and confirmed a large pericardial effusion (Fig. 1b).

Laboratory analyses indicated anemia, leukocytosis with severe lymphopenia, thrombopenia, hyponatremia, hyperkaliemia, a slightly elevated CRP and acute renal failure.

The diagnosis of obstructive shock caused by a large pericardial effusion was made and the patient was transferred to a reference hospital for treatment. On admission to the emergency service of this hospital a percutaneous pericardial drainage was rapidly employed to withdraw 1250 ml of purulent liquid with subsequent normalization of blood pressure. A computed tomography revealed multiple pulmonary, hepatic and splenic septic emboli, as well as lobar pulmonary embolism with bilateral thrombosis of the ilio-femoral veins. A transthoracic echocardiography showed a normal ventricular function and no valvular vegetations or other endocarditis echography signs. The pulmonary pressure was normal and there was no interatrial shunt at the color Doppler. The patient was then started on empirical Piperacillin-Tazobactam and Vancomycin and admitted to the ICU. Blood culture drawn at admission and culture of the pericardial effusion showed a Methicillin-sensitive *Staphylococcus aureus* and antibiotherapy spectrum was narrowed with administration of Flucloxacillin only.

**Fig. 2:**
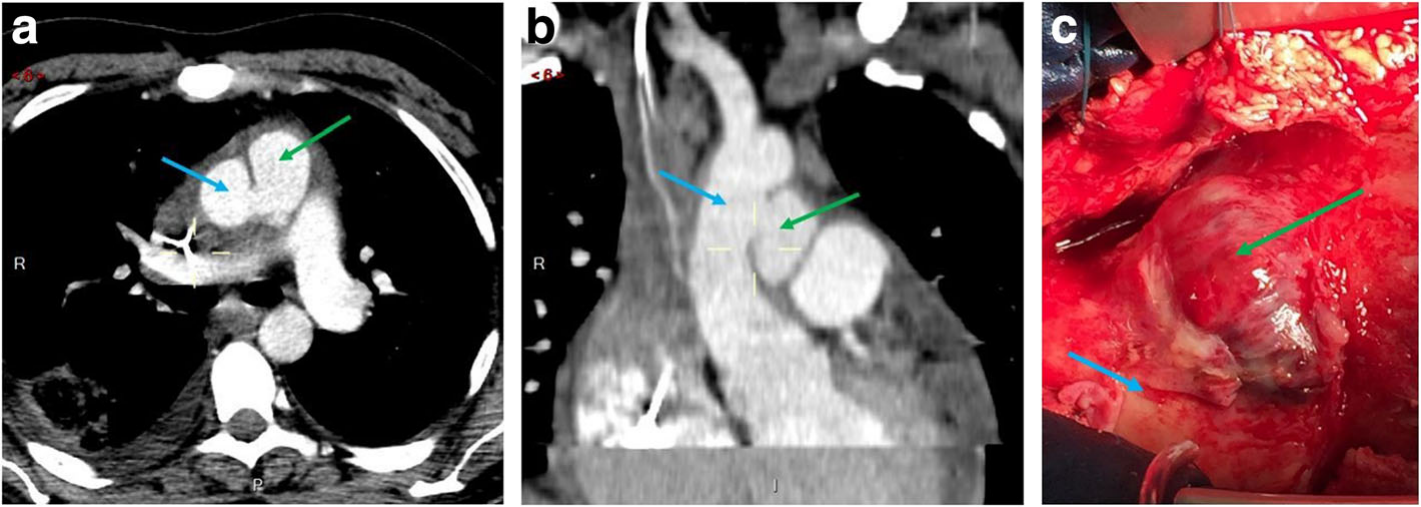
**a** and **b** computed tomography scan views of the false aneurysm (green arrow) and its relationship with the aorta (blue arrow). **c** per-operative view of the aneurysm (green arrow) and the aorta (blue arrow). Patient’s head is oriented towards left of the picture

After 3 days, a follow-up transthoracic echocardiography showed a persistent circumferential pericardial effusion motivating a surgical pericardial drainage through a sub-xiphoidal midline approach. Approximately 1 week later (hospitalization day 10), a thoracic computed-tomography was repeated because of persistent fever and showed a false aneurysm of the ascending aorta measuring 2.5 × 3.7 cm, with signs of aortitis (Fig. 2a and b), as well as multiple new septic emboli. Given the risk of rupture associated with the development of the aneurysm in only 10 days, the patient was rapidly taken to the operating room (Fig. 2c) and a replacement of the ascending tubular aorta and anterior hemiarch using a 26 mm Dacron prosthesis was performed under cardiopulmonary bypass with systemic circulatory arrest in moderate hypothermia at 28 °C and antegrade cerebral perfusion through the bracchio-cephalic arterial trunk.

The patient survived the operation and recovered quite well. He accepted to take an antiretroviral therapy and was started on Truvada and Tivicay. Thrombosis phenomenon in the legs was treated with Rivaroxaban. After a few more days in our hospital the patient was discharged to a regional hospital near his home. The Flucloxacillin therapy was supposed to be pursued for 6 weeks after surgery, but as the patient decided to leave hospital without medical consent a few days later, he was discharged on oral Moxifloxacine once a day.

Eighteen months later the patient is doing well and has stopped all drug consumption.

## Discussion

As said in the introduction, while purulent pericarditis and mycotic aneurysms are uncommon, the combination of the two is even rarer. A survey of Pubmed database has shown only 11 case reports of purulent pericarditis associated with mycotic aneurysm of the aorta [1–11]. Table 1 summarizes the major characteristics of these cases.

**Table 1:** Major characteristics of the studies reporting similar cases, IE: Infective Endocarditis, NR: not reported

Author/Year	Patient characteristics (age/sex/)	Predisposing factors	Pathogen	Proof of IE (yes/no)	Treatment (conservative vs. surgical)	Survival (yes/no)
Fitzgerald 1964 [1]	52/male	None	*S. aureus*	NR	Conservative	no
Brahan 1990 [2]	70/female	Diabetes	C. Septicum	no	Surgical	yes
Shroyer 1990 [3]	65/male	Not reported	S. Aureus	NR	Conservative	no
Aranda 1998 [4]	63/male	None	S. Aureus	no	Conservative	yes
Akashi 2000 [5]	61/female	Chemotherapy	Group B Streptococcus	no	Surgical	yes
Schneider 2004 [6]	60/female	None	S. Enteritidis	no	Surgical	no
Patel 2006 [7]	20/male	None	MRSA	no	Surgical	yes
Saito 2009 [8]	66/female	None	MRSA	no	Conservative	no
Parikh 2009 [9]	46/female	IV drug, hep C, diabetes	*S. agalactiae*, *C. glabrata*	NR	Conservative	yes
Nagano 2010 [10]	76/female	None	S. Pneumoniae	NR	Surgical	yes
Sayah 2013 [11]	70/female	Lung transplant	Scedosporium	NR	Conservative	no

Almost two thirds of patients were female in their 60s and, *Staphylococcus* and *Streptococcus* species were found in eight of the 11 cases. More surprisingly, unlike our patient, more than half of the cases had no known predisposing condition for infective endocarditis. Mortality rate was 45% (5/11) and was essentially seen in patients treated conservatively. This is not surprising as infected aneurysms are best managed with combined treatment including antibiotics and surgery. Unlike in ours, where pericardial effusion appeared first, in a substantial part of the cases pericardial effusion occurred through a retrograde mechanism from the infected aneurysm.

Interestingly, despite repeated transthoracic and transoesophagal echocardiography, no evidence of endocardial involvement could be found as our patient continued to develop new septic emboli. This was also the case in most of the previously published cases. Nevertheless, according to Duke’s criteria, the most probable diagnostic remains infective endocarditis, although an alternative explanation could be paradoxical embolization of septic thrombosis of the legs through a patent foramen ovale.

Moreover, this case underlies the high potential for complications of bacterial infections around the heart. Indeed, in this case, the false aneurysm of the aorta is likely a complication of the purulent pericarditis and the probably underlying endocarditis. Thus, in case of bloodstream infections, especially with high risk pathogens (such as *Salmonella* and *Staphylococcus* species), clinicians must keep a high degree of suspicion regarding potential complications such as mycotic aneurysms, which can be rapidly deadly if not detected in time.

## Conclusion

In summary, we report here an unusual case of purulent pericarditis caused by *S. aureus* infection, which was complicated by the development, in a very short period of time, of a false aneurysm of the ascending aorta, despite an appropriate antibiotic treatment. The condition was successfully managed by a combination of medical and surgical treatment.

## Abbreviations

ICU: Intensive care unit
IE: Infective Endocarditis
POCUS: Point of care ultrasound
